# Lane Detection Method with Impulse Radio Ultra-Wideband Radar and Metal Lane Reflectors

**DOI:** 10.3390/s20010324

**Published:** 2020-01-06

**Authors:** Dae-Hyun Kim

**Affiliations:** ICT Based Public Transportation Research Team, Korea Railroad Research Institute, Uiwang 16105, Korea; daehyunkim@krri.re.kr; Tel.: +82-31-460-5790

**Keywords:** lane detection, impulse radio, UWB (ultra-wideband), radar, metal lane, lane departure warning

## Abstract

An advanced driver-assistance system (ADAS), based on lane detection technology, detects dangerous situations through various sensors and either warns the driver or takes over direct control of the vehicle. At present, cameras are commonly used for lane detection; however, their performance varies widely depending on the lighting conditions. Consequently, many studies have focused on using radar for lane detection. However, when using radar, it is difficult to distinguish between the plain road surface and painted lane markers, necessitating the use of radar reflectors for guidance. Previous studies have used long-range radars which may receive interference signals from various objects, including other vehicles, pedestrians, and buildings, thereby hampering lane detection. Therefore, we propose a lane detection method that uses an impulse radio ultra-wideband radar with high-range resolution and metal lane markers installed at regular intervals on the road. Lane detection and departure is realized upon using the periodically reflected signals as well as vehicle speed data as inputs. For verification, a field test was conducted by attaching radar to a vehicle and installing metal lane markers on the road. Experimental scenarios were established by varying the position and movement of the vehicle, and it was demonstrated that the proposed method enables lane detection based on the data measured.

## 1. Introduction

An advanced driver-assistance system (ADAS) assists a driver in dangerous situations that may not always be recognized by a human driver or a self-driving system. Among the many functions of ADAS, the lane departure warning system (LDWS) and lane keeping assistant system (LKAS) help in safe driving by assisting the driver in case of an unintended departure from the lane. Although the LDWS is a warning system, the LKAS is a more advanced system that has the ability to maintain the lane through steering wheel control in conjunction with the control system. A system that assists in lane keeping helps tremendously in accident prevention and is an essential function for autonomous driving technology in the future.

The most critical factor in successful implementation of any lane keeping technology is lane detection. Currently, the most widely used sensor for lane detection is a camera. Lane detection technology using cameras has been mainly studied to increase its recognition rate in complex environments [1,2,3,4] and to reduce the complexity for real-time lane recognition [5,6,7,8]. However, when cameras are affected by factors, such as lighting conditions, fog, and obstacles, the lane recognition rate is degraded. Furthermore, for the advancement of autonomous driving technology, which has recently gained much attention, it should be able to detect lanes even when the camera is not working well. Therefore, research is needed on lane detection technology using other sensors to complement the camera.

In addition to cameras, light detection and ranging (LiDAR) systems and radars have also been proposed as sensors for lane recognition [9,10,11,12,13]. The method using LiDAR uses the difference in intensity of the received signal reflected from the asphalt and the lane of the road. Although relatively accurate, the cost of implementing a LiDAR system is still high, and it requires a large amount of computation for data processing; hence, it is mainly limited to being used in research on autonomous vehicles [9,10,11]. In a radar-based system, as there is no difference between the reflected signals of asphalt and a lane, a radar reflector is installed in the lane for guidance. Clarke et al. [12] studied the imaging of reflectors on roads with synthetic aperture radars (SARs). Radar signals for reflectors were generated by modeling and not based on actual data, and simulations showed that images of reflectors can be generated. Stolz et al. [13] also investigated installation of metal reflectors along the lanes to detect them with radar. They analyzed the advantages and disadvantages of reflectors by comparing the radar cross-sections with different types of reflectors. For lane recognition, a method of extracting a line by clustering signals close to the line among the detected radar signals is proposed. In general, previous studies on lane recognition using radar have used long-range radar that can have a range of up to tens of meters. In reported studies, simulations or experiments were conducted in an environment without surrounding objects. However, in the practical environment in which a vehicle operates, there are many interference sources around the vehicle such as pedestrians, buildings, and other lanes that reflect signals. The vehicle radar may know the reflected signal strength, direction, and speed information of the detected target, but this information is not enough to distinguish between lanes and other objects. In general, as there is no other object between the vehicle and the lane, minimizing the detection distance to the lane can prevent the interference source from being received by the radar. However, vehicle radars have poor range resolution, making it difficult to distinguish roads and lane reflectors at close range. Difficulties in lane detection with sub-10 m range were also observed in the study by Stolz et al. [13]. Therefore, radar with high-range resolution are needed to distinguish lane and road surfaces at close range.

As discussed above, cameras play a major role in lane detection. However, in a real road environment, there arise some situations wherein lane detection via cameras is difficult such as when the lighting changes suddenly, when direct sunlight hits the lens, or when there is fog. Hence, radar and LiDAR were investigated for lane detection. Even though LiDAR works well with rapid lighting changes, lane detection is still difficult in fog or in direct incident sunlight. In such environments, radar is a good alternative sensor. Radar is slightly ineffective while detecting the position of the lane in comparison with cameras or LiDAR, but it can at least confirm the existence of a lane; thus, it can be used to detect lanes in situations where cameras or LiDAR are difficult to operate. In this study, we propose a new lane detection method using impulse radio ultra-wideband (IR-UWB) radars to contribute to the determination of lane departure in situations where camera and LiDAR do not function well. The IR-UWB radars have a high-range resolution, because they transmit short pulses at the nano- or picosecond [14]. The high-range resolution of IR-UWB radars has been applied to various fields such as detection, location estimation, tracking, recognition, and bio-signal measurement. There have been studies for detecting, estimating, and tracking humans [15,16,17,18,19,20] and further studies for detecting multiple humans [21,22,23]. They are also used in the field of cognition, where they have been used for studies on distinguishing human body movements including hand gestures and mouth movements [24,25,26,27,28]. In addition, they have also been studied to measure bio-signals for detecting movements such as human breathing and heartbeats in a non-invasive manner [29,30,31,32,33,34,35,36,37,38,39]. However, to the best of our knowledge, there has been no research using IR-UWB radars for lane recognition in the automotive field.

In this paper, we propose a lane detection method based on IR-UWB radar with characteristics that are suitable for near-lane recognition. Lane recognition requires the presence of objects with a higher reflectivity than the road surface. In our proposed method, metal plates with the same width and length as the lane markings were installed at regular intervals over the actual lane markings, while the radar was directed toward the road surface. The thickness of the metal plates does not affect the radar reflection, so they could be very thin. However, they should be sufficiently robust to withstand being repeatedly run over by vehicles. In addition, most metals are excellent radar signal reflectors with a higher reflectance being advantageous for lane detection. To prevent noise signals from other than the lane, the detection range was set to a distance where the lane was expected to be detected and the lane reflection energy was in the detection range. The detected energy was a signal reflected from the road surface and the metal lane, and the lane candidate group was selected based on the energy difference. The energy detector output of the lane reflector installed at regular intervals repeated the strength and weakness of the signal periodically. The lane interval was calculated using the estimated period and vehicle speed information, and finally, the existence of lanes was determined. A field experiment was performed to verify the proposed method. To this end, a radar was attached to the rearview mirror of the vehicle and metal lanes were installed at regular intervals and lengths in the same position as the existing lanes. Experimental scenarios were established, and actual data were acquired according to the movement of the vehicle in the lane. Based on the measured data, the proposed method successfully detected lanes and verified whether lanes can be maintained or departed.

## 2. System Model

**Figure 1:**
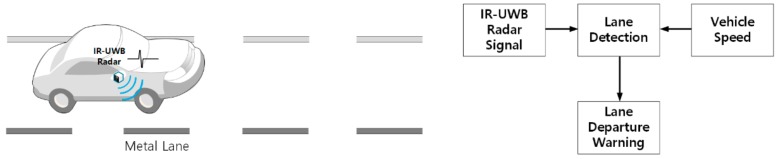
Metal lane detection system model using impulse radio ultra-wideband (IR-UWB) radar.

The proposed lane recognition system model using IR-UWB radar is shown in Figure 1. The IR-UWB radar is installed in the rearview mirror of the vehicle and emits a transmission signal toward the road surface. If the lane reflection signal does not interfere with the vehicle surface reflection, the radar position does not affect the application of the lane detection method proposed herein. On the road, metal lane reflectors (“metal lanes” hereafter) are installed at regular intervals along the lanes and at regular intervals and lengths in the same position as the existing paint markings. The IR-UWB radar transmits impulse signals and receives signals reflected by the road surface and metal lanes. The process of transmitting and receiving an impulse is called a radar scan, and the received signal is an IR-UWB signal. The signal of the *k*th radar scan is given as follows when the transmission pulse *x*(*t*) is received as $N_{k}$ reflected signals:

(1) $$r_{k}\left(t\right)=\overset{N_{k}}{\underset{i=1}{\sum }}a_{ki}x\left(t-t_{ki}\right)+n\left(t\right)$$

where k is a slow time index, which means a single radar scan, and t is a fast time index, the time in which the pulse signal is reflected back after being emitted from the radar, and the value is linearly related to the distance from the radar to the reflector. $a_{ki}$ and $t_{ki}$ are the amplitude and time of arrival of the ith received signal in the kth radar scan, respectively. $n\left(t\right)$ is the observation noise of the UWB radar channel. The proposed lane detection method receives the IR-UWB signal and vehicle speed as input signals. Vehicle speed can be obtained through the on-board diagnostics terminal of the vehicle or by installing sensors such as GPS and odometer. The proposed lane detection method can be applied independently to the left and right lanes of the vehicle. This study focused on the right lane, but the same method can be applied to the left lane, because there is no significant difference in the environments of the right and left lanes. With the proposed lane detection method, it is possible to check whether there are lanes on the left and right sides of the vehicle, so it can be linked with the LDWS.

## 3. Lane Detection Method

**Figure 2:**
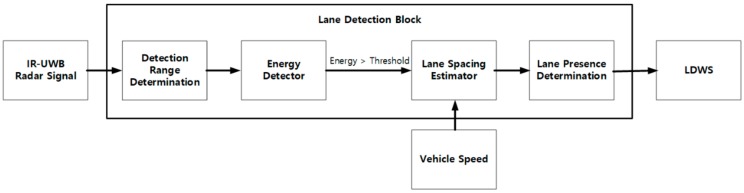
Lane detection method. LDWS: lane departure warning system.

In this section, we discuss the workflow for lane detection shown in Figure 2 to detect metal lanes. The detection range determination block determines the minimum and maximum distances to perform energy detection on the IR-UWB signal. If the detected energy is above the threshold, the process proceeds to the next step, lane spacing estimator block, which calculates the lane interval based on vehicle speed data and the energy detection result over several hours. The lane presence determination block determines whether a lane exists by comparing the estimated lane spacing with the actual lane spacing.

### 3.1. Detection Range Determination and Energy Detector

**Figure 3:**
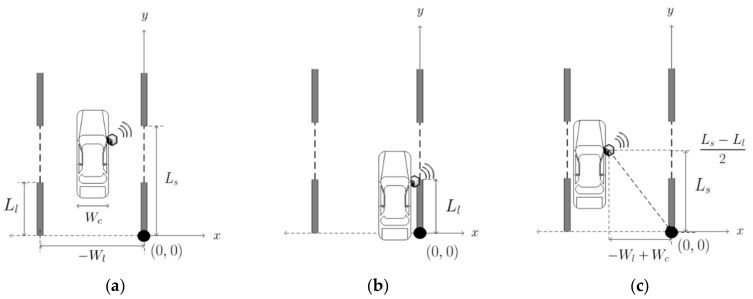
Distance from radar to lane in the *x*, *y* plane: (**a**) parameters required for calculation; (**b**) example at minimum; and (**c**) example at maximum.

The IR-UWB radar signal $r_{k}\left(t\right)$ represents the intensity of the reflected signal according to the distance from the radar. In general, as the position of the target is not known, it is estimated by targeting places where the received signal strength is large. However, the lane detection system model proposed herein predicts the minimum and maximum distances from the radar to the metal lanes if the vehicle starts in the lane. Therefore, the range of energy detection can be set from the minimum to the maximum distance at which the lane is expected to be detected, and the lane can be detected using the magnitude or change of the energy value. The distance calculation from the radar to the lane requires a three-dimensional coordinate system from the plane of x, y to the height of the z-axis, but the height of the radar does not change according to the movement of the vehicle. Consider the x and y coordinate planes as shown in Figure 3a. Let the length of one metal lane be $L_{l}$, the distance between the starting points of two consecutive metal lanes, the lane spacing $L_{s}$, the width of the left and right lanes $W_{l}$, and the vehicle width $W_{c}$. Although not shown in Figure 3, let us assume that the height of the radar is $z=H_{r}$. Figure 3b shows an example where the distance to the radar and lane is the closest. In this situation, the radar is immediately above the lane, i.e., the radar and lane have the same *x* and *y* coordinates (*x* = 0 and $0\leq y\leq L_{l},L_{s}\leq y\leq L_{s}+L_{l}$). For the above coordinates, the distance between the radar and lane is minimal, such that the minimum distance $d_{min}$ can be written as

(2) $$d_{min}=H_{r}$$

Figure 3c shows an example of the longest distance between the radar and lanes when the radar is $\left(x,y\right)=\left(-W_{l}+W_{c},\frac{L_{s}-L_{l}}{2}\right)$ and the lane is $\left(x,\;y\right)=\left(0,\;0\right)$. Therefore, the maximum distance between the radar and lane $d_{max}$ can be written as:

(3) $$d_{max}=\sqrt{{\left(-W_{l}+W_{c}\right)}^{2}+{\left(\frac{L_{s}-L_{l}}{2}\right)}^{2}+H_{r}{}^{2}}$$

The $d_{max}$ value also refers to the minimum distance that the radar should detect; this should be taken into account when selecting a radar. As $d_{min}$ and $d_{max}$ are the distance between the radar and the lane, we can convert them to the time, which is the energy detector input value equal to the time when the round trip distance is moved at the speed of light. Therefore, the minimum and maximum detection ranges $t_{min}$ and $t_{max}$ can be written as:

(4) $$t_{min}=2\times d_{min}/c,$$

(5) $$t_{max}=2\times d_{max}/c$$

where c is the speed of light. If energy detection is performed for the detection range $t_{min}\leq t\leq t_{max}$, the energy detector output $E_{k}$ for the kth scan can be written as:

(6) $$E_{k}=\overset{t_{max}}{\underset{t=t_{min}}{\sum }}{\left|r_{k}\left(t\right)\right|}^{2}$$

When the range resolution of the IR-UWB radar is smaller than the size of the detection object, the optimal detector is the square sum of the received signal when the signal is reflected from multiple range cells of the object [14]. In the proposed system, the IR-UWB radar has a resolution of several centimeters and the lane is several meters in size, so the above energy detector is the optimal detector for this lane detection system. If the detector output value is greater than a threshold, the lane candidate can be detected. The threshold value can be determined by measuring the energy detector output values with and without metal lanes. The threshold determination is discussed in detail in Experimental Results section.

### 3.2. Lane Spacing Estimation and Lane Presence Determination

**Figure 4:**
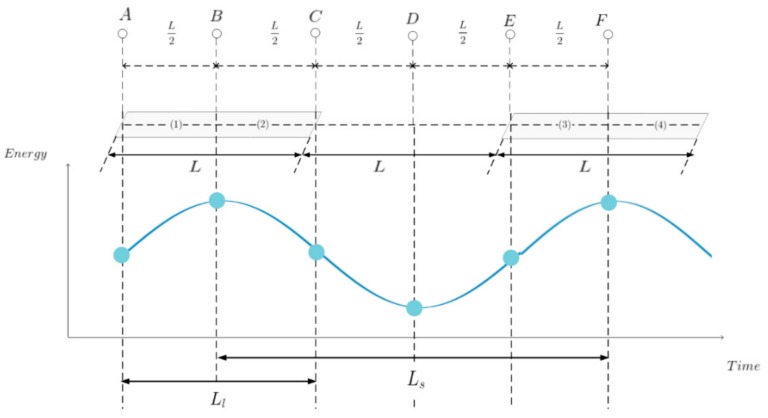
Reflected signal energy according to vehicle and metal lane location.

To estimate lane spacing, let us look at the change in reflected energy as the vehicle moves over two consecutive metal lanes. For simplifying the figures and equations, let $L_{s}=2L$ and $L_{l}=L$ in Figure 4. Suppose the radar moves from y=0 to y=2L with x=0 and $z=H_{r}$ fixed in the coordinate system of Figure 3. Suppose the detection range is from −2L to 2L only in the y-axis direction at each point. As shown in Figure 4, let us consider the result of detecting the energy reflected from the lane at each point, assuming that the position of the radar changes from *A* to *F* as the vehicle moves. Let $E_{A}$, $E_{B}$, $E_{C}$, $E_{D}$, $E_{E}$, and $E_{F}$ be the energies detected by the radar at points *A*–*F*, respectively. To compare the magnitude of the energy reflected by the lanes, divide each lane in half and divide it into four areas, from 1 to 4. The energy reflected from the lanes of the 1st and 2nd regions is detected from −2*L* to +2*L* from the point *A* to the *y*-axis, and the energies of the areas 1 and 2 detected at the point *A* are called $E_{A1}$ and $E_{A2}$, respectively. Similarly, at points *B* and *C*, only the energies reflected in areas 1 and 2 are detected. At point *D*, the energy reflected from areas 2 and 3 is detected, and at the points *E* and *F*, the energy of the signal reflected from the lanes of areas 3 and 4 is detected. This can be summarized and rewritten as:

$$E_{A}=E_{A1}+E_{A2}$$

$$E_{B}=E_{B1}+E_{B2}$$

(7) $$E_{C}=E_{C1}+E_{C2}$$

$$E_{D}=E_{D2}+E_{D3}$$

$$E_{E}=E_{E3}+E_{E4}$$

$$E_{F}=E_{F3}+E_{F4}$$

The reflected energy of area 1 at point *A*, areas 1 and 2 at point *B*, area 2 at point *C*, area 3 at point *E*, and areas 3 and 4 at point *F* are the same. As the distances from the radar to the lanes are the same and the area of each area is the same, the reflected energy is also detected. Therefore, we can write:

(8) $$E_{A1}=E_{B1}=E_{B2}=E_{C2}=E_{E3}=E_{F3}=E_{F4}$$

In addition, the distances from point *A* to area 2, from point *C* to area 1, from point *D* to areas 2 and 3, and from point *E* to area 4 are equal, and the area of areas 1–4 is the same. The energy of the realm is the same and can be written as:

(9) $$E_{A2}=E_{C1}=E_{D2}=E_{D3}=E_{E4}$$

Comparing the value of Equation (8) with the value of Equation (9), the areas 1–4 have the same area, but Equation (8) is closer to the lanes than Equation (9). Therefore, the energy value of Equation (8) is larger than Equation (9) and the following relationship holds:

(10) $$E_{A1}=E_{B1}=E_{B2}=E_{C2}=E_{E3}=E_{F3}=E_{F4}>\;E_{A2}=E_{C1}=E_{D2}=E_{D3}=E_{E4}$$

Substituting the result of Equation (10) into Equation (7), the energy values at each point from *A* to *F* have the following order of magnitude:

(11) $$E_{B}=E_{F}>E_{A}=E_{C}=E_{E}>E_{D}$$

Based on Equation (11), the energy values detected at each point as the radar moves from *A* to *F* are shown in the energy versus time graph at the bottom of Figure 4. The maximum energy value appears in the middle of the first and second lanes and the distance between them is $L_{s}$. The distance $L_{s}$ traveled from *B* to *F* using the time taken to travel between the points *B* and *F* and the speed information of the vehicle can be estimated as follows:

(12) $$\tilde{L_{s}}=\frac{v_{c}\left(t_{F}-t_{B}\right)}{f_{s}}$$

where $f_{s}$ is the radar scanning rate in scans per second, $t_{B}$ and $t_{F}$ are the slow time index at *B* and *F*, respectively, and $v_{c}$ is the vehicle speed. For convenience of explanation, *x* = 0 is assumed, but the radar position changes to $0\leq x\leq -W_{l}+W_{c}$ if the vehicle moves without leaving the lane when the actual vehicle is driven. This is because the vehicle does not move completely straight and there is a lateral movement when moving in the lane. Even in this case, if the energy is compared in the manner above, the tendency to detect the highest energy at the middle point of the lane and the least energy at the middle point between the lanes will not change, but an estimation error $e=\left|L_{s}-\tilde{L_{s}}\right|$ occurs. In the lane presence determination block, it is possible to determine whether a lane exists by determining whether the estimated lane interval that can be recognized as a lane is smaller than the maximum estimated error. Estimation errors can be determined based on data measured in the real environment and are explained in the Experimental Results section.

Next, the range of the values of the parameters mentioned in Figure 3 are described. Assuming that at least N radar scans should be performed for one lane to ensure that the energy detector output represents the periodicity illustrated in Figure 4, the length of one lane $L_{l}$ should satisfy the inequality $V_{c}\times N/f_{s}\leq L_{l}$; then, the minimum value of $L_{l}$ is $V_{c}\times N/f_{s}$. Further, to fully observe one cycle in the energy output curve in Figure 4, the minimum distance $L_{s}$ must satisfy $L_{s}\geq 2L_{l}$; thus, the minimum value of $L_{s}$ is $2L_{l}$. The larger the value of $L_{l}$, the longer the maximum energy value at points B or F. Likewise, the larger the value of $L_{s}$, the longer the minimum energy at point D. Therefore, the larger the values of $L_{l}$ and $L_{s}$, the longer the period of energy detector output value and the longer the lane detection time. Therefore, the optimal lane spacing value is $L_{s}=2L_{l}$ so as to update the lane detection results as soon as possible. The combined width $W_{l}$ of the left and right lanes and the width $W_{c}$ of the vehicle can be seen as constants and depend on the road conditions and the vehicle; hence, they are not parameters that can be varied. Finally, the height $H_{r}$ where the antenna is installed, must be determined in consideration of $W_{l}$, $W_{c}$, $L_{s}$, and $L_{l}$ such that $d_{max}$ in Equation (3) is smaller than the minimum detection distance of the radar.

## 4. Experimental Results

**Figure 5:**
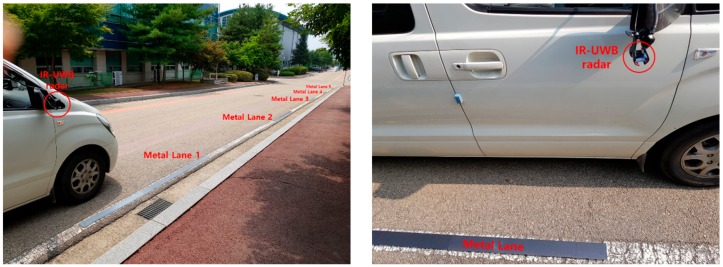
Lane detection field experiment environment.

In order to verify the method proposed above, metal lanes and radars were installed, and field tests were performed as shown in Figure 5. In terms of equipment, only the right lane markings were installed with metal lanes and the radar was installed only on the right side rearview of the vehicle. As there was no significant difference between the right and left side in the road environment, there were no problems in the feasibility verification of the proposed lane detection method. Environment variables were measured as follows: length of the metal lane $L_{l}$ = 3 m, distance between the center of each metal lane (lane spacing) $L_{s}$ = 8 m, width of the lane $W_{l}$ = 3.5 m, vehicle width $W_{c}$ = 1.9 m, radar installation height $H_{r}$ = 1.32 m, and radar scanning rate $f_{s}$ = 20 scans per second. The vehicle traveled at speeds ranging from 20 to 30 km/h (5.56–8.33 m/s), depending on driving conditions. The radar used in this experiment was Xethru X4m03 IR-UWB radar which had a center frequency of 8.748 GHz and a bandwidth of 1.5 GHz. For the above environment, the energy detection range was determined as $d_{min}$ = 1.32 m and $d_{max}$ = 3.65 m by applying Equations (2) and (3). 

**Figure 6:**
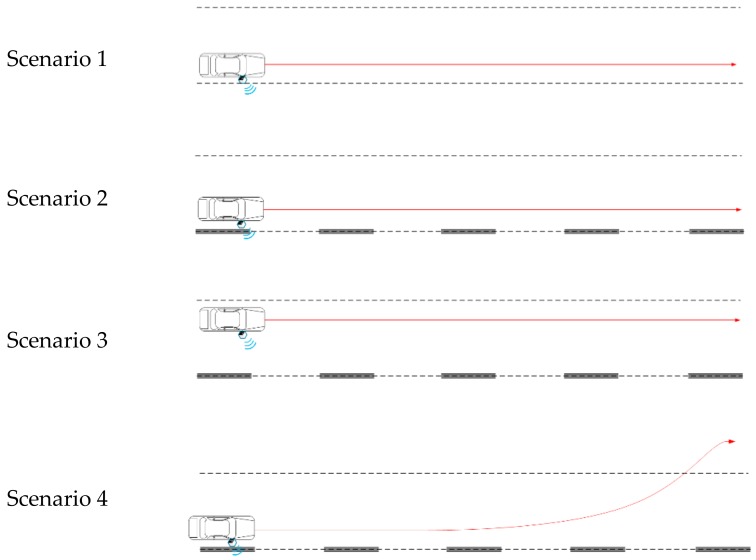
Experiment scenarios.

To test the lane detection method, four scenarios were established by varying the installation of metal lanes and vehicle movement path as shown in Figure 6. Scenario 1 is an environment in which no metal lanes or special reflectors were installed on the road. The vehicle traveled in a straight line, 30 cm from the right lane. In Scenarios 2–4, metal lanes were installed. The vehicle moved at distances of 30 and 120 cm from the right lane markers in Scenarios 2 and 3, respectively. In Scenario 4, the vehicle moved at a distance of 30 cm from the right lane markers between metal lanes 1 and 4, and the vehicle moved from metal lanes 4 to 5 and went over into the lane on the left, from metal lane 4 to 5.

**Figure 7:**
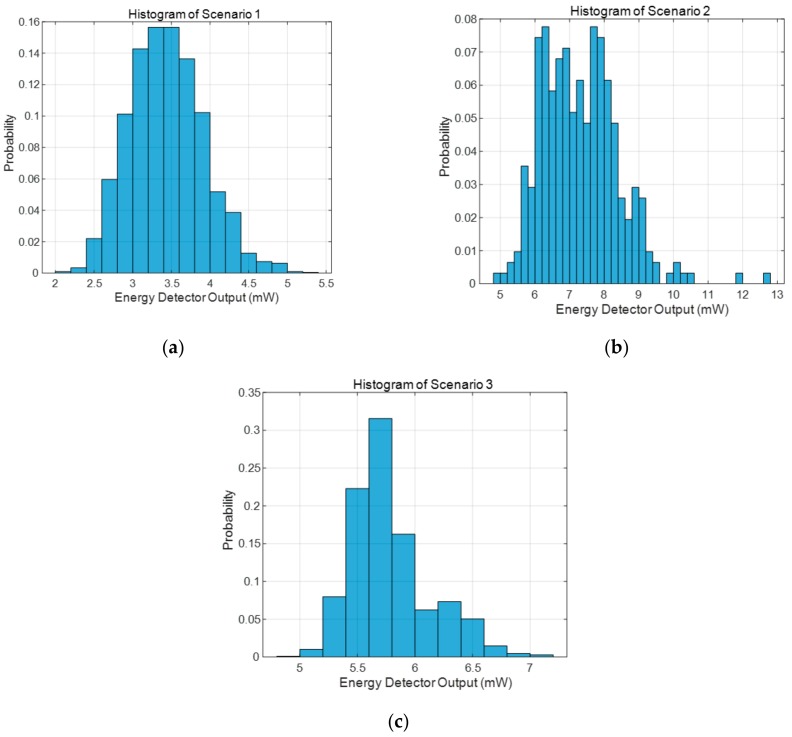
Energy detector output histogram: (**a**) Scenario 1; (**b**) Scenario 2; and (**c**) Scenario 3.

The next step was to determine the threshold value to distinguish lanes and roads by the energy detector output value. For this purpose, energy detector output values were collected through experiments with and without metal lanes. The histogram was plotted by measuring approximately 6000 energy detector output values at different locations between the vehicle and the radar while the vehicle was in the lane. Figure 7a–c are histograms of the energy detector outputs for Scenarios 1, 2, and 3, respectively. Threshold-based decision making is best to minimize the false alarm rate. The proposed method estimates the location of the next lane by considering the vehicle speed based on the previous lane; if it finds the lane above the threshold, a false alarm may occur; hence, the speed-based lane estimation algorithm cannot operate well due to the misidentified lane. Therefore, in the proposed lane recognition method, the threshold was set to 5.4 mW which is larger than 5.36 mW, the maximum value of the energy detector output in Figure 7a; thus, the false alarm rate was 0%. Thus, the metal lane detection rates for Scenarios 2 and 3 were 98.7% and 90.9%, respectively. 

**Figure 8:**
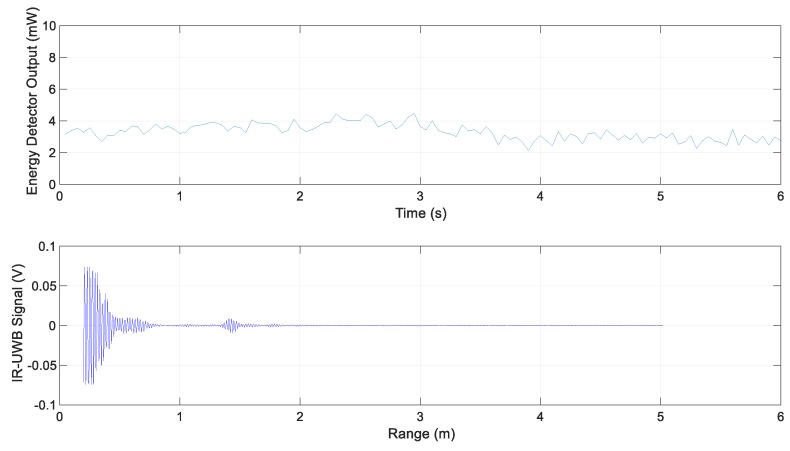
Energy detector output and IR-UWB signal for Scenario 1.

The following are the experimental results for each scenario. The energy detector output for Scenario 1 and the IR-UWB signal at 3.9 s are shown in Figure 8. It represents the energy reflected by simply the road surface without special reflectors; its value was less than the threshold value 5.4 mW.

**Figure 9:**
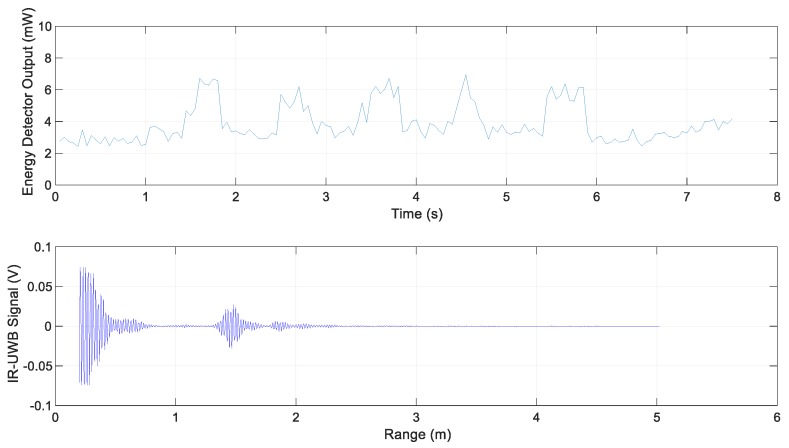
Energy detector output and IR-UWB signal for Scenario 2.

The energy detector output for Scenario 2 and the IR-UWB signal at 1.6 s at the maximum energy in metal lane 1 are shown in Figure 9. The energy detector output shows the highest value as it passes through the center of each metal lane with the energy detector output between 6 mW and 7 mW.

The travel time, speed, and estimated metal lane spacing among maximum energy value points for each metal lane are shown in Table 1. The metal lane installation interval was 8 m and metal lanes were detected with an error of ±0.25 m. The experimental Scenario 2 was the best environment for lane detection, where we obtained a detection rate of 94% and an average lane spacing error of 22 cm by repeating Scenario 2 ten times.

**Figure 10:**
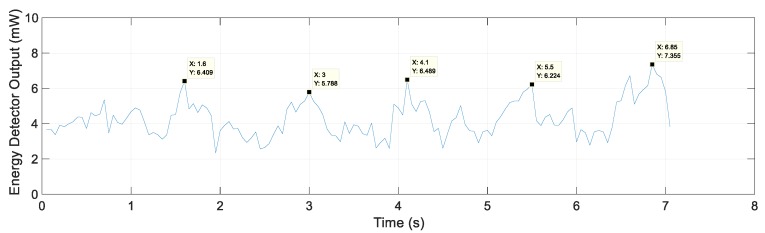
Energy detector output for Scenario 3.

Figure 10 shows the energy detector output for Scenario 3. However, unlike Scenario 2, the energy portion of the lane is unclear, but the lanes were detected above the threshold, and the lane spacing estimate was <20 cm except for lanes 2 and 3, with an error of up to 97 cm for lanes 2 and 3 as shown in Table 2. The experimental Scenario 3 was the worst environment for lane detection, where we obtained a detection rate of 86% and average lane spacing error of 38 cm by repeating Scenario 3 ten times.

**Figure 11:**
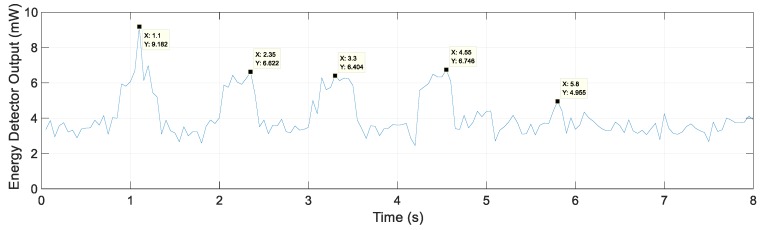
Energy detector output for Scenario 4.

Scenario 4 is when a vehicle moves from metal lane 4 to 5 and goes over into the lane on the left. In Figure 11, metal lanes 1 to 4 were detected, and the energy detector output had the highest value of 4.96 mW at 5.8 s and did not exceed the threshold value of 5.4 mW; hence, the metal lane 5 was not detected. In this scenario, if a vehicle leaves the lane, the lane is not detected, and a warning signal is sent to the LDWS. Metal lane spacing estimates for Scenario 4 are shown in Table 3. 

**Figure 12:**
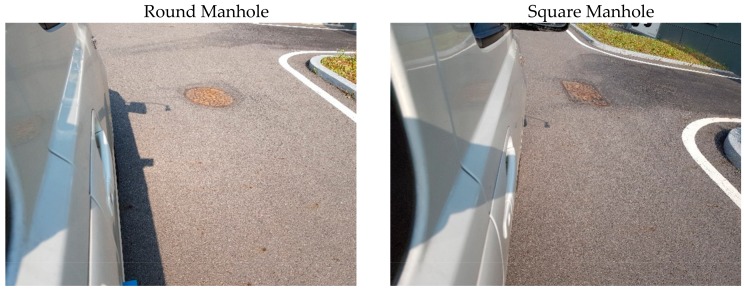
Manhole measurement environment.

**Figure 13:**
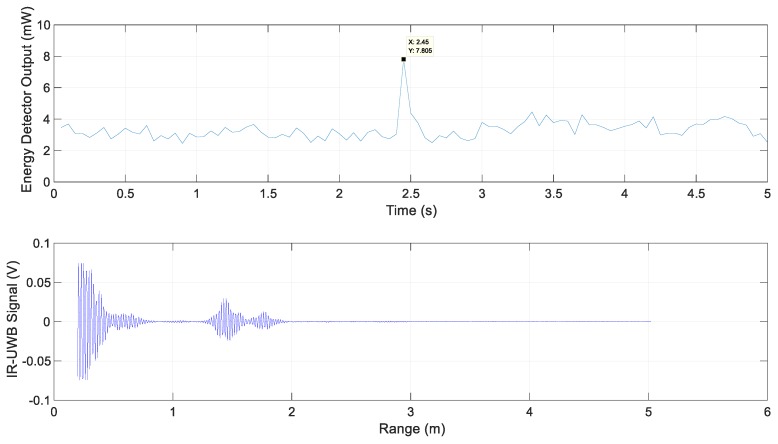
Energy detector output and IR-UWB signal for round manhole.

**Figure 14:**
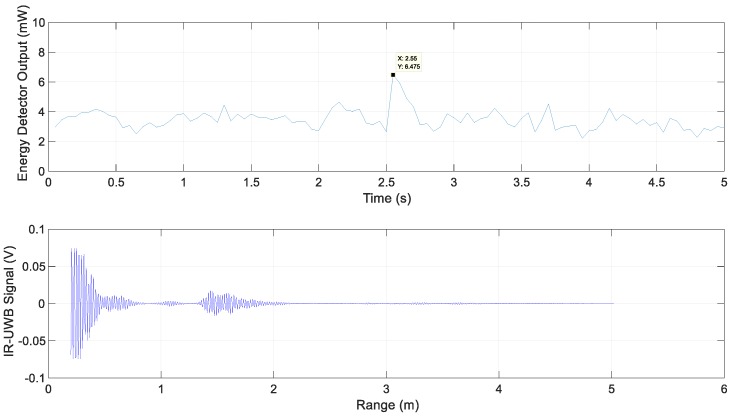
Energy detector output and IR-UWB signal for square manhole.

Finally, the reflection signal by the manhole that could be mistaken as a metal lane among the metallic facilities on the road was examined. The round and square manholes were tested, and the manholes used for the measurement are shown in Figure 12. The received signal and energy detector output for the round and square manholes are shown in Figure 13 and Figure 14, respectively. Both manholes were detected with an energy detector output of 5.4 mW or more but would not be detected as lanes, because they do not appear in series with periodicity.

## 5. Conclusions

An IR-UWB radar-based lane detection method was proposed and verified through experiments. Metal lane markers were installed because radar cannot be used to recognize common paint lane markers. The proposed method estimates lane spacing using the periodic changes in reflected energy values in the regularly installed lane and by comparing it with the actual value to determine a lane. This result can be used in conjunction with the LDWS to assist drivers in an environment where camera-based lane recognition technology does not operate smoothly or to improve the autonomous driving recognition technology. In addition, the proposed lane recognition system can also be utilized for estimating the travel distance or position through counting of lane markers. The results from field experiments showed that the obtained lane detection rates were approximately 86%–94% for the experimental scenarios and 90.9%–98.7% in the sample collection step for the histogram. This difference was because the experiment was performed by precisely moving the vehicle at a speed of 10 km/h in the sample collection step, whereas the vehicle moved at 20–30 km/h in the experimental scenarios and slightly swayed toward the left and right. This difference may also be attributed to the difference in the number of samples. The lane spacing estimation error was in the range of 22–38 cm on average; however, actual errors occurred up to 97 cm. The reason for the large error in the lane spacing estimation was that the vehicle did not move in a direction that was completely parallel to the lane. However, this type of vehicle swaying is encountered at all times, even during actual vehicle driving scenarios. This error can be compensated for if the inertial sensor information or the steering information of the vehicle can be used. Accurate lane spacing estimates will help eliminate the sources of high reflectivity such as manholes.

In this study, the possibility of lane detection using IR-UWB radar was demonstrated. Although this method is difficult to apply to all roads owing to the installation cost of metal lanes, it can be applied at places where cameras encounter difficulty while operating such as tunnel entrances and exits, places that are prone to fog, and places with direct sunlight. As radars must also be installed, our proposed model may be appropriate for vehicles requiring high safety, such as public transportation, rather than for all vehicles. The areas for further study toward commercialization are as follows. First, the energy detector output must be modeled by accumulating and analyzing experimental data in various road environments such as curved roads and sloped roads. To increase the lane detection rate, research on lane detection-specific IR-UWB and antenna radiation pattern optimization is also required.

## Figures and Tables

**Table 1:** Metal lane spacing estimation for Scenario 2.

Metal Lane Numbers	1–2	2–3	3–4	4–5
Travel Time (s)	1.1	1	0.95	1.1
Vehicle Speed (m/s)	7.22	7.78	8.33	7.5
Estimated Lane Spacing (m)	7.94	7.78	7.91	8.25

**Table 2:** Metal lane spacing estimation for Scenario 3.

Metal Lane Numbers	1–2	2–3	3–4	4–5
Travel Time (s)	1.4	1.1	1.4	1.35
Vehicle Speed (m/s)	5.83	6.39	5.83	5.83
Estimated Lane Spacing (m)	8.16	7.03	8.16	7.87

**Table 3:** Metal lane spacing estimation for Scenario 4.

Metal Lane Numbers	1–2	2–3	3–4	4–5
Travel Time (s)	1.3	1	1	1.15
Vehicle Speed (m/s)	7.22	7.78	7.78	7.5
Estimated Lane Spacing (m)	9.39	7.78	7.78	8.63
